# Seniors and Undergraduates Mutually Benefit From an Interprofessional Service-Learning Health Promotion Program

**DOI:** 10.1093/geroni/igaa057.025

**Published:** 2020-12-16

**Authors:** Britteny Howell

**Affiliations:** University of Alaska Anchorage, Anchorage, Alaska, United States

## Abstract

Although benefits of service-learning and interprofessional education (IPE) have been separately well documented to be effective for students in gerontology and geriatrics courses, few curricula appear to integrate both aspects into a single course for undergraduate students in public health. This poster discusses the development and implementation of a service-learning health promotion program utilizing IPE embedded within two courses in two different departments at a mid-sized university. Students worked in interdisciplinary teams and acquired interprofessional educational learning outcomes while they engaged in their first experiences working with diverse older adults at a low-income, independent-living housing community. Twenty-five students (N=25) each team-taught 2 sessions on nutrition, physical activity, and stress reduction techniques in a 10-week program. Qualitative and quantitative results are presented which demonstrate significant learning outcomes from the students about the health needs of the aging population and increased comfort in working with older adults. Older participants in the program also reported positive health and psychological outcomes from their participation. Limitations, challenges, and next steps are also presented.

